# Bladder Spasm Discomfort After Transurethral Surgery: A Prospective Observational Study of Preoperative, Intraoperative, and Postoperative Predictive Factors

**DOI:** 10.7759/cureus.84508

**Published:** 2025-05-20

**Authors:** Titos Markopoulos, Stamatios Katsimperis, Lazaros Lazarou, Lazaros Tzelves, Iraklis Mitsogiannis, Athanasios Papatsoris, Andreas Skolarikos, Ioannis Varkarakis

**Affiliations:** 1 Urology, Second Department of Urology, National and Kapodistrian University of Athens, Athens, GRC; 2 Urology, Second Department of Urology, National and Kapodistrian University of Athens, Sismanogleio General Hospital, Athens, GRC

**Keywords:** bladder spasms, catheter-related bladder discomfort, postoperative pain, transurethral resection of bladder tumor, transurethral resection of the prostate, turbt, turp

## Abstract

Catheter-related bladder discomfort (CRBD) is a common and distressing complication following transurethral urologic procedures such as transurethral resection of the prostate (TURP) and transurethral resection of bladder tumors (TURBT). This prospective observational study investigated the role of preoperative, intraoperative, and postoperative factors in predicting the severity of postoperative bladder spasms. A total of 122 patients were enrolled, and bladder discomfort was assessed using the Visual Analogue Scale (VAS) during their postoperative hospital stay. Most clinical and surgical variables, including anesthesia type, procedure type, catheter type, energy modality, and patient demographics, showed no significant association with bladder discomfort severity. However, catheter balloon volume emerged as a significant predictor, with patients receiving 40 mL balloon volumes reporting higher VAS scores compared to those with smaller volumes (p = 0.003). Additionally, a weak but statistically significant correlation was found between hematocrit drop and VAS scores (rho = 0.18, p = 0.047), suggesting a possible link between intraoperative blood loss and postoperative discomfort. These findings highlight the potential for simple interventions, such as optimizing catheter balloon volume, to alleviate CRBD and enhance postoperative recovery.

## Introduction

Catheter-related bladder discomfort (CRBD) is a frequent and distressing complication in patients undergoing transurethral surgeries such as transurethral resection of the prostate (TURP) and transurethral resection of bladder tumors (TURBT) [1]. CRBD encompasses a range of symptoms including suprapubic pain, urinary urgency, and involuntary bladder spasms [2,3]. These symptoms can negatively affect recovery, increase analgesic consumption, and reduce overall patient satisfaction.

The pathophysiology of CRBD is primarily attributed to involuntary detrusor contractions mediated by muscarinic receptor activation in the bladder wall, particularly the M3 subtype [4]. The presence of an indwelling catheter, along with bladder irrigation and balloon pressure, can trigger this response by stimulating afferent nerve fibers, leading to increased acetylcholine release and detrusor overactivity [5]. Notably, the location of the catheter balloon at the bladder trigone is thought to aggravate symptoms, especially at higher inflation volumes.

CRBD incidence varies depending on surgical procedure, patient characteristics, and catheter-related factors. Studies have reported CRBD rates ranging from 47% to 90% following TURP and TURBT [6-8]. Despite the high prevalence, there remains limited understanding of modifiable predictors of postoperative bladder spasms. Pharmacological management options include antimuscarinics (e.g., oxybutynin, tolterodine), gabapentinoids, nonsteroidal anti-inflammatory drugs (NSAIDs), and intravenous lidocaine [9-13]. Additionally, non-pharmacologic approaches such as pudendal nerve blocks, transcutaneous electrical nerve stimulation and catheter optimization strategies are gaining interest [14-17].

From a healthcare systems perspective, CRBD contributes to increased nursing workload, prolongs hospital stay, and can increase the likelihood of unplanned readmissions [18]. Given its impact on quality of life and resource utilization, identifying modifiable predictors is critical. This study aimed to assess the impact of preoperative, intraoperative, and postoperative variables on bladder spasm severity in patients undergoing transurethral urologic procedures.

## Materials and methods

This prospective observational study enrolled patients between the ages of 25 and 90 years who underwent TURP or TURBT. The protocol initially aimed for 200 patients, but final inclusion was based on the availability of complete data and informed consent, resulting in a cohort of 122 patients. Data extraction commenced in 2018 and concluded in 2024. Patients were recruited from a tertiary care hospital. Exclusion criteria included the presence of neurologic conditions affecting bladder function, spinal cord pathology, psychiatric disorders, chronic cystitis (chemical, interstitial, or radiation-induced), and use of a pigtail catheter. Ethical approval was obtained from the institutional review board (Protocol Number: 22046/24.10.18), and written informed consent was secured from all patients prior to participation. All procedures were performed by experienced urologists. Catheterization protocols followed standardized institutional guidelines, with balloon volumes adjusted based on surgeon preference and case specifics.

Data collection included patient demographics, clinical history, and perioperative details. Preoperative variables included age, gender, American Society of Anesthesiologists (ASA) score, International Prostate Symptom Score (IPSS), and prostate volume (via ultrasound). Intraoperative data encompassed type of anesthesia, operation type, surgical duration, energy modality (monopolar vs. bipolar), and disease characteristics. Postoperative parameters included catheter type (two-way vs. three-way), bladder irrigation, post-void residual urine (PVR), analgesia administered, and catheter balloon volume.

Bladder spasm intensity was assessed using the Visual Analogue Scale (VAS) daily during the postoperative hospital stay. Patients rated their discomfort on a scale from 0 (no discomfort) to 10 (severe discomfort). All VAS scores were recorded by trained nursing staff during morning and evening shifts to ensure consistency.

Statistical analyses were performed using R (R statistical software, Vienna, Austria). Descriptive statistics included means with standard deviations (SD) and medians with interquartile ranges (IQR). Normality of distributions was assessed using Shapiro-Wilk tests and by visual inspection of box plots and histograms. Non-parametric comparisons were performed using the Wilcoxon rank-sum and Kruskal-Wallis tests. The Kruskal-Wallis test was applied to compare VAS scores across different balloon volume groups and provided a single H statistic and overall p-value for the comparison of all groups. Post-hoc pairwise comparisons were conducted to identify specific group differences, with p-values adjusted accordingly. Spearman correlation coefficients were calculated for continuous variables. A p-value less than 0.05 was considered statistically significant.

## Results

A total of 122 patients (75 males, 47 females) were included. The mean patient age was 69.8 years (SD±11.9). Among them, 82 underwent TURBT and 40 underwent TURP. The average operative duration was 29.6 minutes (SD±15.6). Epidural anesthesia was administered to 110 patients and general anesthesia to 12 patients. In terms of catheter type, 52 patients (42.6%) had two-way catheters and 70 (57.4%) had three-way catheters. Monopolar resection was performed in 66 patients (54.1%) and bipolar resection in 56 patients (45.9%). The baseline characteristics of the patients are presented in Table 1.

**Table 1:** Baseline characteristics of patients SD: standard deviation, TUR-BT: transurethral resection of bladder tumor, TUR-P: transurethral resection of prostate

Variable	Sample (n) or Mean (SD)
Age (years)	69.8 (11.9)
OR time (min)	29.6 (15.6)
Gender	
Males	102 (83.6%)
Females	20 (16.4%)
Type of energy used	
Monopolar	66 (55%)
Bipolar	56 (45%)
Type of operation	
TUR-BT	82 (67.2%)
TUR-P	40 (32.8%)
Catheter type	
2-way	52 (42.6%)
3-way	70 (57.4%)
Type of anesthesia	
General	12 (9.8%)
Epidural	110 (90.2%)

The median VAS score for bladder discomfort was 2 (IQR 1-4). Of the total cohort, 56 patients (46%) reported mild discomfort (VAS 0-2), 40 (33%) had moderate discomfort (VAS 3-5), and 26 patients (21%) experienced severe discomfort (VAS ≥6). Subgroup comparisons revealed no statistically significant differences in VAS scores by type of anesthesia (epidural vs. general, p=0.51), procedure (TURBT vs. TURP, p=0.36), catheter type (p=0.21), energy modality (p=0.22), or gender (p=0.56). These comparisons are summarized in Table 2.

**Table 2:** Comparison of VAS scores Results are presented as medians (25th-75th percentile values) of VAS scores. Test statistics are from Wilcoxon rank-sum tests (Mann-Whitney U). W-statistics are reported. All p-values are two-tailed; significance threshold set at p = 0.05. VAS: Visual Analogue Scale, TUR-BT: transurethral resection of bladder tumors, TUR-P: transurethral resection of prostate

Variable	Group 1	Group 2	Test statistic	p-value
Type of anesthesia				
Epidural	2 (1-4)	2 (1-4)	W = 0.0	0.51
General	2.5 (1-5)	2.5 (1-5)	W = 0.0	0.51
Type of operation				
TUR-BT	2 (1-4)	2 (1-4)	W = 0.0	0.36
TUR-P	3 (2-4)	3 (2-4)	W = 0.0	0.36
Type of energy used				
Monopolar	2 (1-3)	2 (1-3)	W = 0.0	0.22
Bipolar	3 (1-5)	3 (1-5)	W = 0.0	0.22
Catheter type				
2-way	2 (0-4)	2 (0-4)	W = 50.0	0.21
3-way	2 (1-4)	2 (1-4)	W = 50.0	0.21
Gender				
Male	2 (1-4)	2 (1-4)	W = 100.0	0.56
Female	1.5 (0-5)	1.5 (0-5)	W = 100.0	0.56

Spearman correlation analysis showed no significant associations between VAS scores and age (rho = 0.04, p = 0.69), IPSS (rho = -0.02, p = 0.82), or prostate volume (rho = 0.12, p = 0.23). Hematocrit drop showed a weak but statistically significant correlation with VAS score (rho = 0.18, p = 0.047), suggesting a potential relationship between intraoperative blood loss and postoperative bladder discomfort. Correlation coefficients and significance values are shown in Table 3.

**Table 3:** Correlation between VAS scores and patient variables Test statistic is Spearman’s rank correlation coefficient (rho). VAS: Visual Analogue Scale, IPSS: International Prostate Symptom Score

Variable	rho	Test statistic	p-value
Age	0.04	Spearman	0.69
IPSS	-0.02	Spearman	0.82
Prostate volume	0.12	Spearman	0.23
Hematocrit drop	0.18	Spearman	0.047

**Figure 1:**
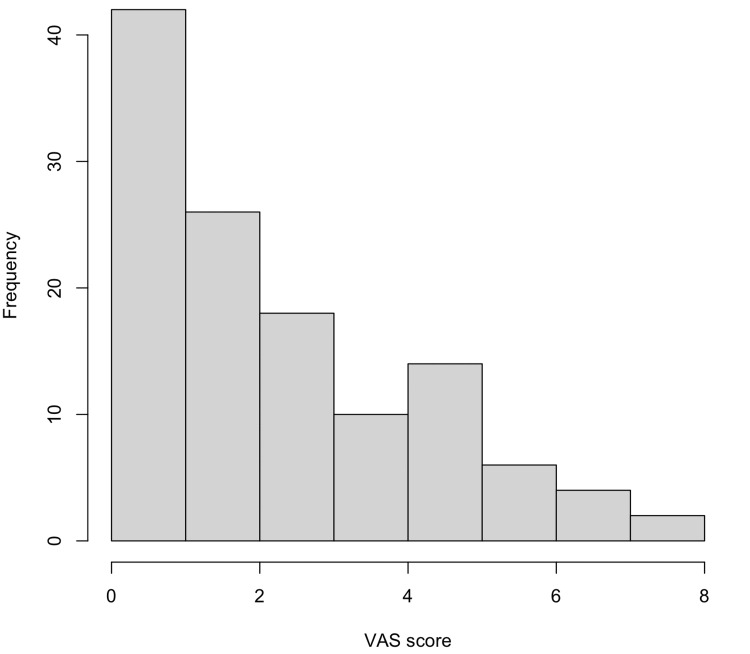
Histogram showing the distribution of Visual Analog Scale (VAS) scores for bladder discomfort in the study population (n = 122). The distribution is right-skewed, supporting the use of non-parametric statistical analysis.

Catheter balloon volumes ranged from 10 to 50 mL, with the most common volumes being 20 mL (n=42), 30 mL (n=38), and 40 mL (n=18). Patients with 40 mL balloon volume reported significantly higher median VAS scores compared to other volume groups (p = 0.003) (Figure 1). No significant differences were noted among the remaining balloon volume groups. There was no institutional protocol standardizing balloon volume, allowing for natural variation based on surgeon preference. Detailed comparisons across balloon volume groups are presented in Table 4.

**Table 4:** Comparison of VAS scores according to saline infused in catheter balloon Sample size (n) is shown for each balloon volume group. Test statistics derived from Kruskal-Wallis test comparing VAS scores across groups. Significant difference observed at 40 mL (p = 0.003). VAS: Visual Analogue Scale, IQR: interquartile range

Balloon Volume (mL) (n)	Median VAS Score (IQR)	Test Statistic	p-value
10 (20)	2 (1–3)	H = 1.2	0.79
15 (2)	2 (1–4)	H = 1.5	0.79
20 (68)	2 (1–4)	H = 0.9	0.99
35 (2)	3 (2–5)	H = 1.0	0.99
40 (24)	4 (3–6)	H = 12.6	0.003
50 (6)	3 (1–5)	H = 1.3	0.99

**Figure 2:**
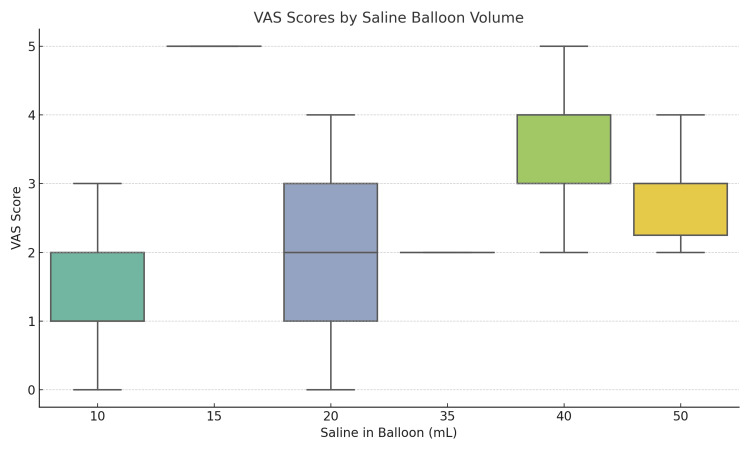
Box plot showing the distribution of Visual Analog Scale (VAS) scores according to catheter balloon volume. Patients with 40 mL balloons reported significantly higher discomfort (p = 0.003).

Analgesic use was not systematically recorded in this study and thus could not be correlated with discomfort scores. No missing data were identified during analysis. The distribution of VAS scores was right-skewed, supporting the use of non-parametric tests (Figure 2). Data integrity was confirmed through manual validation and double-entry verification.

## Discussion

This study investigated clinical and perioperative factors associated with bladder spasm severity following transurethral surgery. Consistent with recent studies, most patient characteristics and surgical variables examined in this study were not significantly associated with postoperative discomfort, despite the wide range of factors evaluated [19]. The weak but significant correlation between hematocrit drop and VAS scores suggests that blood loss, surgical trauma or prolonged surgery may increase the risk of discomfort, potentially due to greater bladder wall irritation or inflammation. While this association was modest, it warrants further exploration in larger cohorts. The most striking finding was the strong association between catheter balloon volume and VAS score. Specifically, patients with 40 mL balloon volumes reported higher levels of discomfort, supporting the hypothesis that increased intravesical pressure exacerbates CRBD symptoms. These results align with the known pathophysiology of CRBD, which is driven by muscarinic receptor-mediated bladder contractions in response to bladder wall and trigone irritation [4,20,21]. The foreign body nature of the catheter, combined with the mechanical pressure from the balloon, can provoke involuntary spasms, particularly when the volume is excessive. Prior studies have shown that reducing balloon volume or optimizing catheter design can reduce CRBD symptoms [17,20]. Binhas et al. further studied the role of Foley catheter size as a predictive factor for bladder spasms, demonstrating that discomfort increases notably when the catheter size exceeds 18F, especially in men [22]. Although our study did not focus on treatment strategies, the results have clinical relevance. Catheter management, including optimizing balloon volume, may represent a low-cost intervention to improve patient comfort. While pharmacologic treatments such as antimuscarinics, lidocaine, and gabapentinoids remain central to management, mechanical factors should not be overlooked [17]. Additionally, findings support the development of individualized postoperative care protocols based on simple modifiable factors. CRBD places a burden not only on patients but also on clinical care teams. Nursing staff are often required to provide additional analgesia, manage catheter complaints, and respond to increased patient agitation [18]. In some cases, severe discomfort may lead to catheter dislodgement or premature removal, compromising procedural outcomes or necessitating re-catheterization.

Limitations of the study include its observational design, which limits the ability to establish causality. The study was conducted in a single healthcare setting, potentially limiting generalizability. Bladder spasm assessment relied on patient-reported outcomes using VAS scores, which are subjective by nature. Additionally, the study did not include long-term follow-up, assess the effect of pharmacological interventions, or record analgesic use. Future research incorporating objective measures, such as urodynamic studies, and randomized comparisons of catheter techniques may provide further insights.

## Conclusions

Bladder spasms following transurethral urologic procedures represent a common and distressing postoperative complication. This prospective study highlights that while a variety of patient demographics and surgical variables were assessed, most showed no significant association with postoperative bladder discomfort. However, two important modifiable factors - catheter balloon volume and hematocrit drop - emerged as contributors to increased discomfort levels. Notably, patients with higher catheter balloon volumes reported significantly higher VAS scores, suggesting that simple mechanical factors can meaningfully impact postoperative recovery. The weak but significant correlation between hematocrit drop and discomfort further emphasizes the role of surgical precision in minimizing intraoperative trauma. These findings underscore the importance of optimizing catheter management practices, particularly balloon volume, as a practical and low-cost strategy to enhance patient comfort and improve postoperative outcomes. Future efforts to standardize catheter protocols and refine intraoperative techniques may further reduce the burden of catheter-related bladder discomfort in clinical practice.
